# High-intensity focused ultrasound (HIFU) versus deep brain stimulation (DBS) for refractory tremor: team HIFU

**DOI:** 10.1055/s-0045-1809660

**Published:** 2025-07-17

**Authors:** Karina Silveira Massruhá, Ellison Fernando Cardoso

**Affiliations:** 1 Universidade de São Paulo, Faculdade de Medicina, Departamento de Neurologia, Centro de Distúrbios do Movimento, São Paulo SP, Brazil.; 2 Hospital Israelita Albert Einstein, Departamento de Neurologia, São Paulo SP, Brazil.; 3 Hospital Israelita Albert Einstein, Departamento de Imagem, São Paulo SP, Brazil.; 4 Universidade de São Paulo, Faculdade de Medicina, Hospital das Clínicas, Instituto de Radiologia, Seção de Neurorradiologia, São Paulo SP, Brazil.

**Keywords:** Essential Tremor, Parkinson Disease, High-Intensity Focused Ultrasound Ablation, Tremors

## Abstract

High-intensity focused ultrasound (HIFU) has emerged as a minimally invasive and incision-free alternative for managing tremors associated with essential tremor (ET) and Parkinson's disease (PD). Approved by the United States Food and Drug Administration (FDA) for unilateral and staged bilateral thalamotomy, HIFU also addresses cardinal PD symptoms such as rigidity and bradykinesia through pallidotomy. Tremor improvement rates range from 50 to 75% for ET and 60 to 90% for tremor-dominant PD, with long-term efficacy sustained up to 5 years posttreatment, including 73% tremor improvement in a recent controlled multicenter study. Unlike deep brain stimulation (DBS), HIFU eliminates hardware-related complications, such as infections and intracerebral hemorrhage, and minimizes postprocedural maintenance. Adverse events are primarily mild and transient, including temporary paresthesia and imbalance. Real-time magnetic resonance imaging (MRI) guidance enhances targeting precision, enabling patients to resume daily activities within 24 hours. These attributes make HIFU a durable and effective treatment option.

## INTRODUCTION

High-intensity focused ultrasound (HIFU) has emerged as a novel modality for treating tremors associated with essential tremor (ET) and Parkinson's disease (PD). This minimally invasive and incision-free technique has gained traction in both clinical and research settings over the past decade. 1 Although relatively new in its current application, the use of focused ultrasonic waves for biological purposes dates back to 1942, 2 having advanced further in the 1950s with the use of focal lesions. 3

Advances in transcranial sonication transformed HIFU into a minimally invasive procedure, positioning it as a viable alternative to DBS with greater patient acceptance. Approved by the United States Food and Drug Administration (FDA), applications of HIFU in movement disorders have been designed unilaterally for patients with medication-refractory ET 4 and tremor-dominant Parkinson's disease (TdPD). 5 More recently, this technique includes unilateral pallidotomy for treating additional PD symptoms such as bradykinesia, rigidity, and dyskinesias, 6 as well as staged bilateral thalamotomy for treating ET at least 9 months after the initial procedure. 7

## HIGH EFFICACY AND LONG-TERM RESULTS

Studies consistently demonstrate HIFU's high efficacy, with tremor reduction rates for unilateral thalamotomy of 50 to 75% for ET 4 8 9 10 and 60 to 90% for TdPD. 5 11 12 13 Staged bilateral procedure for ET targeting the ventral intermediate (Vim) nucleus of the thalamus demonstrates 66% tremor improvement at 3 months, with sustained results at 6 and 12 months. 7 These outcomes are comparable to unilateral Vim-DBS, where tremor improvement in TdPD ranges from 67% in the short-term to 58% in long-term follow-up (>10 years). For ET, it ranges from 66% in the short term to 48% in the long term (>10 years), with this decline likely attributable to disease progression and the development of “tolerance” to DBS. 14

A systematic review analyzing 45 studies comparing the effectiveness of DBS and HIFU in ET found that bilateral DBS is superior to HIFU for tremor reduction. However, no significant difference was observed between unilateral DBS and HIFU. At a mean follow-up of approximately 14 to 16 months, unilateral DBS improved tremor by 56.4%, while bilateral showed a 61.2% improvement, compared to 55.6% in the HIFU group, with all procedures being performed unilaterally. 15 To date, no studies directly compare bilateral DBS to staged bilateral HIFU for ET tremor improvement.

In PD, asymmetrical motor symptoms and motor fluctuations, including dyskinesias, also respond well to HIFU pallidotomy. A recent multicenter, prospective, double-blind, randomized, sham-controlled trial reported that 69% of patients experienced at least a three-point improvement in the movement disorders society-unified Parkinson's disease rating scale, part III (MDS-UPDRS III, OFF state) or the unified dyskinesia rating scale (UDysRS, ON state). 6 Although not yet FDA-approved for this indication, targeting the subthalamic nucleus (STN) has demonstrated significant improvements in cardinal PD symptoms beyond tremor. Rigidity improved by 60 to 83%, and bradykinesia showed improvements of 33 to 69% in reported studies. 12 13

The long-term outcomes further reinforce the durability of HIFU as a tremor-management strategy. A controlled, multicenter clinical trial with 5 years of follow-up reported sustained tremor improvement of 73%, with overall better in quality of life and disability scores measures, without any progressive or delayed complications. 16 17 18

Establishing and implementing HIFU in low- and middle-income countries with resource-limited settings is considered feasible, with the majority of patients achieving significant clinical improvement and only a minority experiencing transient intra- or postprocedural adverse events (AEs). 19 These findings underscore HIFU as a viable, durable, and effective therapeutic option for tremor management in movement disorders, even in resource-constrained environments.

## MINIMIZED ADVERSE EVENTS

Although some patients are considered good candidates for DBS, approximately 45% are reluctant or unwilling to undergo the procedure. The main reasons include fear of AEs, financial burden, and hope for new nonsurgical treatments. 20

The DBS technique involves invasive neurosurgical implantation, whereas HIFU is an incision-free procedure with no associated “device” related AEs. There were 46 articles describing the outcomes and the adverse effects of unilateral and bilateral Vim-DBS in patients with ET, which found surgical- and device-related incidence of AEs were and 6.4% and 11.5%, respectively. 21 The first group of AEs include infections (3.4%), asymptomatic bleeding (2.9%), intraoperative intracerebral hemorrhage (2.4%), and wound dehiscence (2.6%). The latter group mainly includes lead fracture (5.3%) and lead repositioning (3.8%).

Retrospectively analyzing AEs up to 10 years postoperatively involving 510 cases of DBS for PD, ET, and dystonia, mainly targeting the STN, but also the Vim and the globus pallidus interna (GPi), the incidence of surgical and device related AEs were consistent. In this tertiary movement disorders center, they found the risks include intracerebral hemorrhage (3%), subdural hematoma (1,5%), mental status changes (3%), and hardware-related complications (5%). 22 Additionally, stimulation-related side effects occur in 26,3 to 49% of cases within the 1 ^st^ year and can be limiting factors for its optimal adjustment, although these are typically transient and easily improved by adjusting the parameters. 14 21

In a cohort of 98 patients from an observational study, paresthesia and dysarthria were present in approximately 17% of patients, 14 which is consistent with other studies. 23 These are the most frequent side effects when targeting the Vim nucleus of the thalamus. Gait instability and ataxia are also frequently reported in approximately 10% of patients, being limiting factors for adjusting stimulation parameters. Those findings have been consistently reinforced in a more recent meta-analysis, in which the most common stimulation-related AEs were dysarthria (10.5%), paresthesia (6.3%), hemiparesis/paresis (6.3%), and headache (6.7%). 21

In contrast, HIFU AEs are primarily mild and transient, such as temporary paresthesia or imbalance. 4 5 7 10 Studies have reported that approximately 70 to 85% of AEs are mild. 7 18 Nonetheless, persistent side effects have also been documented, primarily including paresthesia or numbness (17–20%), dysarthria (14%), imbalance (5%), and gait disturbances (5%). 18 Furthermore, long-term follow-up showed no new AEs related to the procedure from 12 months onward, with sustained safety profiles up to 5 years posttreatment. The longest follow-up study on HIFU for ET found no severe AEs after 5 years. 18 Although these findings underscore the favorable safety profile of HIFU, a small subset of patients may experience a decline in initial benefits within 6 to 12 months. Given the irreversible nature of the lesion, clinicians must carefully balance therapeutic efficacy with procedural safety. Notably, in cases where disease progression necessitates additional intervention, DBS electrodes have been successfully implanted in regions previously targeted by HIFU, even up to 4 years after the initial procedure, highlighting the potential for a complementary treatment strategy. 6 7 12 18

The gold standard surgical treatment for medication-resistant ET and PD is still DBS. 14 15 24 25 However, as a relatively novel technique, HIFU presents an alternative approach for treating medication-refractory ET and TdPD. Given that DBS requires ongoing postoperative management, carries hardware-related risks, and may raise concerns regarding surgical invasiveness, a comparison with HIFU is warranted as the latter serves as an effective, minimally invasive alternative for selected patients with movement disorders.

## REAL-TIME PRECISION FOR ENHANCED SAFETY

Real-time magnetic resonance imaging (MRI) guided temperature mapping raises safety and precision during the procedure. Low-power, short duration sonications (10–15 seconds) are first used to visualize thermal changes without tissue damage, ensuring the accurate positioning of the “hot spot”. This step allows for precise lesioning during high-power sonication, thereby reducing the risk of unintended damage.

While DBS may experience intraprocedural challenges such as brain shift caused by cerebrospinal fluid (CSF) leakage, HIFU eliminates this risk by avoiding brain incisions altogether. This lack of invasiveness enhances targeting precision, particularly when combined with continuous clinical evaluations, which can be performed throughout the procedure with the patient awake.

## FASTER RECOVERY AND MINIMAL FOLLOW-UP CARE

Another advantage of HIFU is that it offers immediate tremor relief. Following the procedure, the transducer and head frame are removed, and patients are typically discharged the same day or within 24 hours, allowing them to quickly resume routine activities.

Furthermore, this technique involves minimal postprocedure care, in contrast with DBS, which entails a prolonged recovery period and frequent follow-ups for device programming and optimization. 26 The latter also requires an initial period of programming to achieve optimal therapeutic outcomes, and follow-up visits become less frequent once optimal settings are established, typically occurring three times per year to assess stimulation parameters, potential AEs, disease progression, and tolerance to stimulation in case of ET. Additionally, impedance checks are conducted each time the device is checked. It is essential to ensure that patients are adequately informed about battery replacements, which are generally required every 4 to 5 years, though more frequent replacements may be necessary in cases of higher energy consumption. 25

The streamlined recovery process coupled with its low-maintenance nature makes HIFU a particularly appealing option for patients seeking effective treatment with minimal disruption to their lives. Additionally, it offers significant advantages for individuals living in remote areas with limited access to healthcare or those facing cultural and social barriers to ongoing medical follow-up. 19

## TECHNOLOGY ADVANCES AS EXPERTISE GROWS

Centers where staged bilateral procedures were performed had fewer side effects after the second procedure in comparison to the first, possibly because of the cumulative experience of the treatment team, smaller lesion size, and improvements in target selection, 27 reinforcing that the learning curve of expert teams plays a pivotal role in optimizing outcomes.

Economic analyses conducted in the United Kingdom and Canada support HIFU as a cost-effective treatment for TdPD and ET. Studies have demonstrated that it is significantly more affordable than unilateral DBS, while offering comparable or slightly superior effectiveness. 28 29 However, as a novel technique still in the early stages of clinical implementation, HIFU remains less available and may encounter inconsistent insurance coverage until it becomes firmly established in clinical practice.

Additionally, continuous advancements in technology, such as improved neuroimaging (that is, diffusion-weighted imaging tractography – DTI targeting) for greater target precision and better correlation of procedure parameters with lesion features, will optimize clinical outcomes. 30 31
